# Risk factors for residual pelvic obliquity one year after total hip arthroplasty

**DOI:** 10.1007/s00590-024-04060-z

**Published:** 2024-08-20

**Authors:** Yuto Ozawa, Yusuke Osawa, Yasuhiko Takegami, Hiroki Iida, Genta Takemoto, Shiro Imagama

**Affiliations:** 1 Department of Orthopaedic Surgery, Nagoya University Graduate School of Medicine, 65 Tsurumai, Showa-ku, Nagoya, Aichi 466-8550 Japan; 2 Department of Orthopaedic Surgery, Toyohashi City Hospital, 50 Hakkennishi, Aotaketyo, Toyohashi, 441-8570 Japan

**Keywords:** Pelvis, Pelvic obliquity, Total hip arthroplasty, Perception of leg length discrepancy

## Abstract

**Purpose:**

It is not uncommon for patients with hip disorders to present with pelvic obliquity (PO), and residual PO after total hip arthroplasty (THA) may not only affect hip joint function but also cause adjacent intervertebral joint disorders. This study aimed to investigate the postoperative PO impact on clinical outcomes and risk factors by comparing patients who had PO after THA to those who did not.

**Methods:**

A single-center, retrospective cohort study was conducted. A total of 103 patients who underwent THA were included in this study from 2018 to 2020. Demographics, functional outcomes, and spinopelvic parameters were compared between post-THA PO of less than 2° (NT group, 55 patients) and PO of 2° or more (O group, 48 patients). Multivariate analysis was performed using factors with significant differences in univariate analysis.

**Results:**

Postoperative Harris Hip Score Activity was significantly lower in the T group than in the NT group (p = 0.031). Preoperative PO was smaller in the NT group than in the T group (p = 0.001). Preoperative lumbar bending range (LBR) was significantly more flexible in the NT group than in the T group. In the logistic regression analysis, Age (odds ratio 0.957, 95% CI 0.923–0.993, p = 0.020), preoperative PO (odds ratio 1.490, 95% CI 1.100–2.020, p = 0.001), and LBR (odds ratio 0.848, 95% CI 0.756–0.951, p = 0.005) were found to be significant factors.

**Conclusion:**

Younger age and large preoperative PO, and poor lumbar spine mobility were identified as risk factors for residual postoperative PO.

## Introduction

Pelvic obliquity, defined as an abnormal rotation of the pelvis in the coronal plane, contributes toward adjacent joint and intervertebral joint disorders, such as knee and lumbar osteoarthritis [1–4]. There have been many observed cases of hip osteoarthritis due to pelvic obliquity, and pelvic obliquity has also been associated with the perception of leg length discrepancy (P-LLD) and hip function in patients with osteoarthritis [5]. In this sense, total hip arthroplasty (THA) is a fundamental treatment to improve hip osteoarthritis [6–9]. However, in clinical practice, cases of residual pelvic obliquity after THA have been reported, which may affect implant survival owing to early loosening [10]. Therefore, it is crucial to restore hip function, including pelvic obliquity, to achieve higher satisfaction.

Pelvic obliquity can be classified as suprapelvic, intrapelvic, or infrapelvic according to its etiology [11]. Hip joint contractures of abduction and adduction and cases with P-LLD can cause infrapelvic obliquity, while cases with acetabulum in an abnormal position can cause intrapelvic obliquity [10, 11]; both of which can generally be improved by THA. In contrast, suprapelvic obliquity is caused by scoliosis and osteoarthritis, and it is difficult to improve the suprapelvic factors even after THA [12]. In addition, it has been reported that patients with a reduced lumbar bending range (LBR) and more than 2º of preoperative pelvic obliquity are more likely to have residual P-LLD [13]. However, the factors that influence pelvic obliquity after THA are unclear.

The purpose of this study was (1) to investigate the impact of postoperative pelvic obliquity on clinical outcomes and (2) to identify risk factors for residual pelvic obliquity by comparing patients who had residual pelvic obliquity after THA to those who did not. We hypothesized that residual pelvic obliquity after THA worsens postoperative clinical outcomes and there are some risk factors for residual pelvic obliquity after THA.

## Material and methods

### Patients

**Fig. 1:**
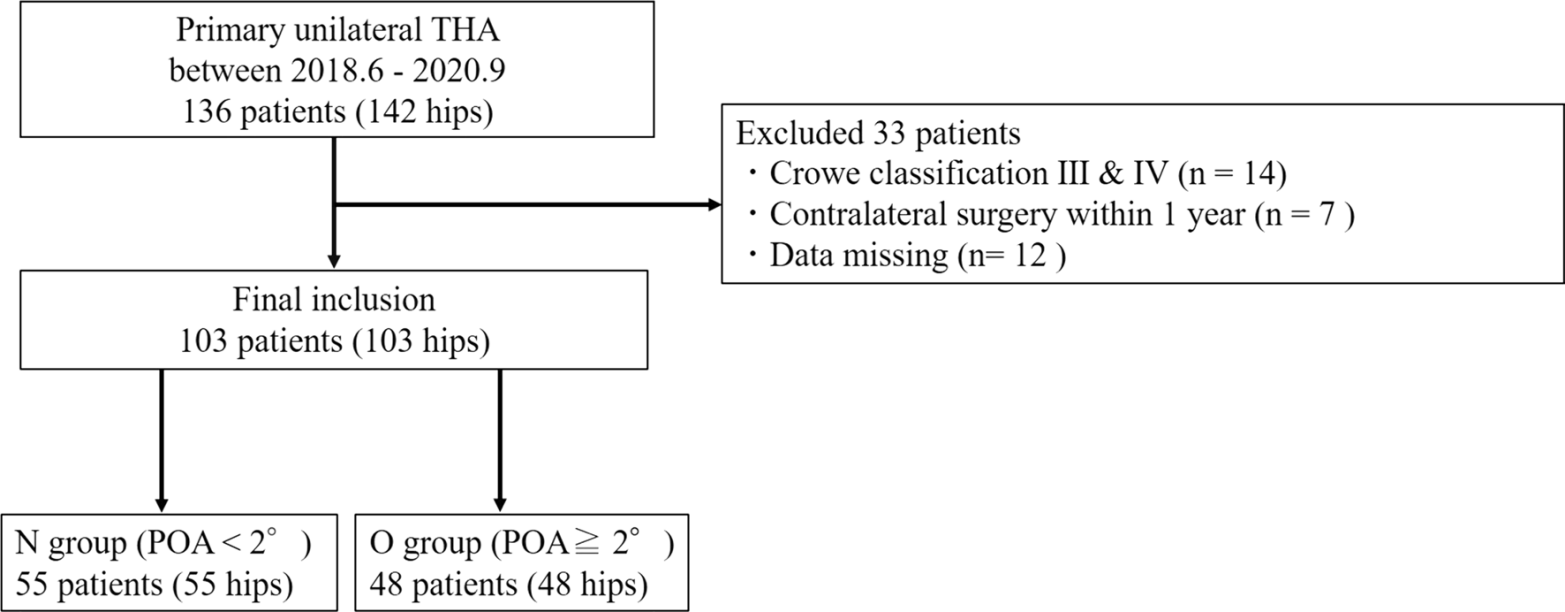
Patient flow-chart

In this retrospective cohort study, 143 patients who underwent unilateral total hip arthroplasty (THA) at our hospital between June 2018 and September 2020 were initially considered. Exclusions totaled 40 cases: 14 in Crowe’s classification groups III and IV, 14 with contralateral surgery within one year, and 12 with insufficient imaging evaluation, leaving 103 cases for analysis (Fig. 1). Based on the report by Koga et al., a case–control study was conducted [13]. According to previous reports, the average lateral PO in healthy individuals is approximately 2° [14]. Therefore, patients were divided into two groups based on the pelvic obliquity angle (POA) measured in the first postoperative year from the anteroposterior radiograph of the patient while standing: those with less than 2° POA (N group, n = 55) and those with more than 2° POA (O group, n = 48). This study was performed under the certification of the Ethics Committee of Nagoya University Hospital.

### Surgical procedure and perioperative management

THA was performed in all cases using the standard posterior approach, with the patient in the lateral decubitus position. Postoperatively, all patients underwent rehabilitation, including ambulation, range of motion (ROM), and muscle training.

Preoperatively, three-dimensional (3D) planning was performed using ZedHip software (Lexi, Tokyo, Japan) to determine the implants, aiming to adjust leg length discrepancy (LLD) and offset to match those of the contralateral side as closely as possible [15]. Implant placement was determined at the surgeon’s discretion. The femoral components included 43 Secur-Fit Advanced (Stryker, Mahwah, NL), 26 Exeter (Stryker, Mahwah, NL), 1 Accolade II (Stryker, Mahwah, NL), 14 Taperloc Microplasty (Zimmer-Biomet, Warsaw, IN), 6 CPT (Zimmer-Biomet, Warsaw, IN), and 13 Initia (KYOCERA, Kyoto, Japan). The acetabular components included 58 Trident (Stryker, Mahwah, NL), 12 X3 RimFit (Stryker, Mahwah, NL), 20 G7 OsseoTi (Zimmer-Biomet, Warsaw, IN), and 13 SQRUM (KYOCERA, Kyoto, Japan). All surgeries were performed by a single senior surgeon or by a junior surgeon under the guidance of a senior surgeon.

### Clinical evaluation

Background data, such as age, sex, body mass index (BMI), hip diagnosis, lateralization, and contralateral hip status, were extracted from the patients’ medical records.

Hip function was assessed using the Harris Hip Score (HHS) and ROM before and one year after surgery. ROM was measured by a senior surgeon using a goniometer. Quantitative measurements of P-LLD were performed according to the method of Koga et al. [12], with and without P-LLD, and the absolute value of P-LLD was evaluated. Additionally, changes in P-LLD (ΔP-LLD) from preoperative to postoperative measurements were recorded. The HHS, ROM, and P-LLD were measured by a senior surgeon.

### Hip radiographic evaluation

**Fig. 2:**
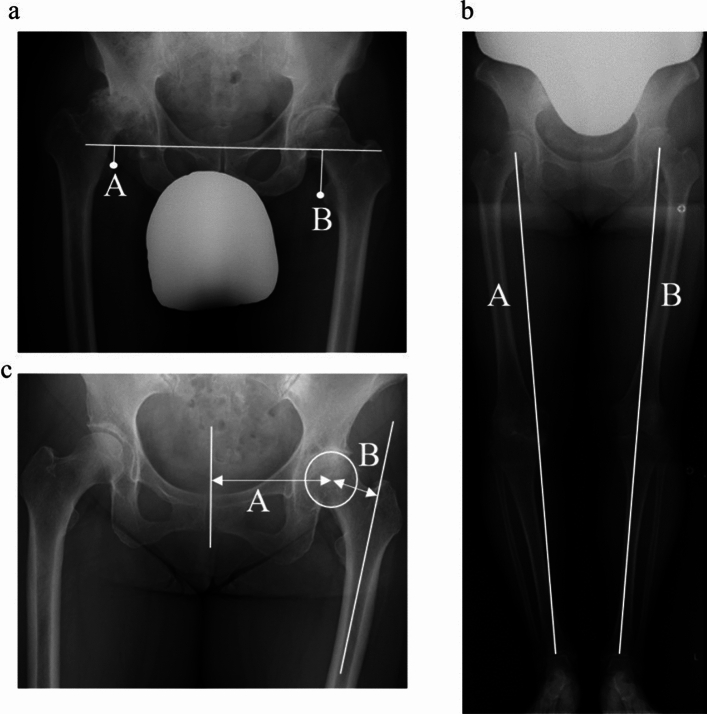
Hip parameters.**a** B – A = Radiographic leg length discrepancy. **b** B – A = Functional leg length discrepancy. **c** A = Acetabular offset, B = Femoral offset

Radiographic evaluation of the hip joint included radiographic leg length discrepancy (R-LLD), functional leg length discrepancy, and femoral, acetabular, and global offset preoperatively and 1-year postoperatively. R-LLD was defined as the difference in vertical distance between the nonoperative and operative sides from the line drawn at the most inferior edge of the bilateral acetabular tear scar to the lesser trochanter (a positive value indicated a shortened hip joint on the operated side; Fig. 2a), according to the method of Woolson et al. [16] Functional leg length discrepancy was defined as the distance from the center of the femoral head to the center of the ankle joint subtracted from the contralateral side by the operated side (a positive value indicated a shortened leg length on the operated side; Fig. 2b) [8]. The femoral offset was the distance from the femoral head center to the femoral shaft; the acetabular offset was the distance from the perpendicular line through the pubic symphysis to the femoral head center; and the global offset was the sum of those two distances (Fig. 2c) [17].

Radiological measurements for R-LLD, functional leg length discrepancy, and femoral and acetabular offset were performed for all 103 cases by two surgeons. The inter-observer reliability values for these measurements were determined to be 0.81, 0.83, 0.88, and 0.85, respectively.

### Spine radiographic evaluation

Radiographic evaluation of the spine and pelvis was performed preoperatively and at 1-year postoperatively for POA, Cervical 7 coronal vertical axis (C7CVA), lumbar scoliosis angle, LBR, and sagittal parameters. In the standing position, anteroposterior and lateral radiographs of the patient were evaluated.

**Fig. 3:**
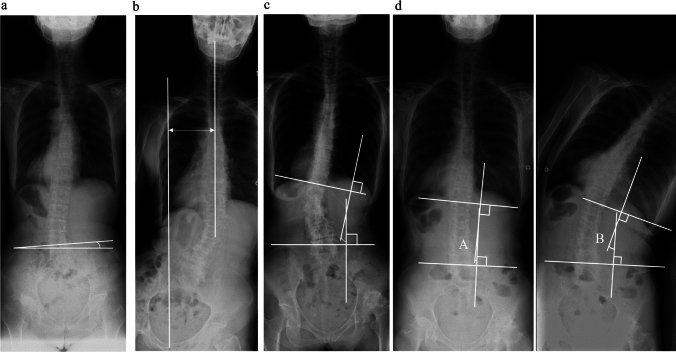
Spinal radiographic parameters. **a** Pelvic obliquity angle (POA). **b** Cervical 7 coronal vertical axis (C7CVA). **c** Lumbar scoliosis angle. **d** Lumber bending range (LBR)

POA was defined as the angle between the horizontal line, tangent to the most proximal iliac crest, following the method of Osebold et al. [18] (POA is expressed as absolute values; Fig. 3a).

C7CVA was measured according to the method of Nakashima et al. [19] and defined as the horizontal distance between the center sacral vertical line (CSVL) and C7 (the positive direction is defined as the operated-side direction; Fig. 3b). The lumbar scoliosis angle was defined as the angle between the line of the superior border of the L1 lumbar vertebra and the line passing through the superior border of both sides of the iliac crest (a positive value was defined as a lumbar scoliosis angle that is convex to the hip joint on the affected side; Fig. 3c) [5]. The angle between the superior aspect of L1 and the Jacoby line was measured in the standing full spine frontal and maximum lateral flexion position according to the method of Koga et al. [13]. The difference between the angle in the standing frontal and maximum lateral flexion to the affected side was defined as LBR (Fig. 3d) [13].

Sagittal parameters measured were pelvic tilt (PT), sacral slope (SS), pelvic incidence (PI), lumbar lordosis (LL), thoracic kyphosis (TK), and C7 sagittal vertical axis (C7SVA). Measurements were performed as previously described [17]. C7SVA was defined as the distance from the central tangent of C7 to the upper sacral end (a positive value was defined when C7 was further forward).

Radiological measurements for pelvic obliquity, C7CVA, lumbar scoliosis angle, LBR, PT, SS, PI, LL, TK, and C7SVA were performed for all 103 cases by two surgeons. The inter-observer reliability for these measurements was determined to be 0.83, 0.82, 0.75, 0.78, 0.83, 0.87, 0.83, 0.80, 0.88, and 0.79, respectively.

### Statistical analysis

Statistical analyses consisted of Student’s t-test for continuous variables and the Chi-square test for categorical variables. Factors affecting postoperative pelvic obliquity were evaluated using logistic regression analyses for items with a p-value < 0.05. Inter-observer reliability was evaluated using intraclass correlation coefficients (ICCs), with one indicating perfect correlation and zero indicating poor correlation. The measurements were performed by two surgeons. Statistical analysis was performed using EZR (Saitama Medical Center, Jichi Medical University), and a p-value < 0.05 was considered statistically significant [20].

## Results

Sex, age, height, weight, BMI, diagnosis, and left, right, and contralateral hip conditions did not differ between the two groups (Table 1). Preoperative HHS and ROM were not different between the two groups, but postoperative HHS activity was significantly lower in the O group (11.4 ± 2.5) than in the N group (12.4 ± 1.9) (p = 0.031). Total HHS was 86.7 ± 12.5 in the O group and 90.5 ± 8.0 in the N group, which was not significantly different, but tended to be lower in the O group (p = 0.068) (Table 2). P-LLD was not different between groups preoperatively; however, postoperatively, the N group was significantly less likely to experience P-LLD compared with the O group (25.5% vs. 58.3%; p = 0.001). The length of P-LLD was also significantly different: 0.37 ± 0.87 cm in the N group and 0.91 ± 0.97 cm in the O group (p = 0.004) ΔP-LLD was not significantly different between the groups. (p = 0.315) (Table 3). The preoperative POA was smaller in the N group (2.5 ± 1.5°) than in the O group (3.8 ± 2.3°) (p = 0.001). No differences in preoperative lumbar scoliosis angle, C7-CVA, PT, SS, PI, LL, PI-LL, TK, and SVA were observed. Preoperative LBR was significantly more flexible in the N group (9.3 ± 4.5°) than O group (5.8 ± 4.7°) (p < 0.001). Postoperative POA was significantly smaller in the N group (0.8 ± 0.6°) than in the O group (4.0 ± 2.0°) (p < 0.001) (Table 4). Preoperatively and postoperatively, functional leg length discrepancy and femoral, acetabular, and global offset were comparable between the two groups. Preoperatively, R-LLD was not significantly different between groups, whereas postoperatively, R-LLD was significantly shorter on the healthy side in the O group (− 3.4 ± 10.3 mm) than in the N group (0.1 ± 7.0 mm) (p = 0.048) (Table 5).

**Table 1:** Patient demographics

	N Group (n = 55)	O Group (n = 48)	p
Sex (male/female)	11/44	11/37	0.811
Height cm (mean ± SD) [min–max]	156.1 ± 8.4 (133.5–174)	155.4 ± 8.6 (138–183)	0.661
Weight kg (mean ± SD) [min–max]	59.0 ± 10.4 (40.4–86.0)	58.2 ± 12.4 (35.5–99.8)	0.742
BMI kg/m^2^ (mean ± SD) [min–max]	24.2 ± 4.0 (16.2–36.4)	24.0 ± 4.1 (15.9–34.4)	0.811
Age at THA (mean ± SD) [min–max]	64.0 ± 12.7 (29–85)	60.3 ± 14.2 (25–84)	0.164
Diagnosis (OA/ONFH)	48/7	42/6	1
Laterality, hips (right/left)	28/27	31/17	0.170
Other side (healthy/THA/OA/ONFH)	28/12/9/6	23/9/11/5	0.875

*BMI*, Body Mass Index; *THA*, Total hip arthroplasty; *OA*, Osteoarthritis; *ONFH*, Osteonecrosis of the femoral head

**Table 2:** Clinical evaluations

	N Group (n = 55)	O Group (n = 48)	p
*HHS (preoperative)*			
Pain (mean ± SD) [min–max]	18.1 ± 7.9 (0–40)	19.3 ± 8.0 (0–44)	0.449
Gait (mean ± SD) [min–max]	20.1 ± 6.2 (8–30)	20.2 ± 6.6 (8–30)	0.997
Activity (mean ± SD) [min–max]	9.0 ± 2.4 (3–14)	8.4 ± 2.5 (3–14)	0.235
Total (mean ± SD) [min–max]	51.0 ± 16.5 (13–78)	52.2 ± 15.6 (28–83)	0.720
*HHS (1 year)*			
Pain (mean ± SD) [min–max]	42.9 ± 2.9 (30–44)	41.9 ± 5.1 (20–44)	0.222
Gait (mean ± SD) [min–max]	28.9 ± 5.5 (17–33)	28.3 ± 6.2 (8–33)	0.603
Activity (mean ± SD) [min–max]	12.4 ± 1.9 (6–14)	11.4 ± 2.5 (3–14)	0.031
Total (mean ± SD) [min–max]	90.5 ± 8.0 (60–100)	86.7 ± 12.5 (31–100)	0.068
*Range of motion (preoperative)*			
Flexion (mean ± SD) [min–max]	86.6 ± 22.7 (25–120)	84.1 ± 25.5 (15–120)	0.589
Extension (mean ± SD) [min–max]	2.2 ± 7.2 (-20–15)	2.0 ± 9.8 (-20–25)	0.904
Abduction (mean ± SD) [min–max]	17.2 ± 10.0 (0–40)	15.0 ± 8.6 (0–40)	0.240
Adduction (mean ± SD) [min–max]	14.5 ± 10.9 (0–30)	13.4 ± 6.5 (0–30)	0.575
External rotation (mean ± SD) [min–max]	21.1 ± 11.4 (-15–45)	20.9 ± 12.5 (0–60)	0.948
Internal rotation (mean ± SD) [min–max]	14.7 ± 16.5 (-20–50)	12.7 ± 15.7 (-10–60)	0.527
*Range of motion (1 year)*			
Flexion (mean ± SD) [min–max]	103.2 ± 18.0 (50–135)	98.6 ± 14.1 (70–135)	0.158
Extension (mean ± SD) [min–max]	7.3 ± 8.0 (-20–40)	7.7 ± 6.6 (-5–20)	0.815
Abduction (mean ± SD) [min–max]	27.5 ± 7.1 (10–40)	25.5 ± 8.3 (5–50)	0.202
Adduction (mean ± SD) [min–max]	13.2 ± 5.5 (0–30)	11.2 ± 4.5 (0–20)	0.086
External rotation (mean ± SD) [min–max]	23.5 ± 11.0 (0–45)	21.9 ± 10.6 (0–50)	0.459
Internal rotation (mean ± SD) [min–max]	31.1 ± 14.2 (5–60)	27.8 ± 13.8 (0–60)	0.235

HHS; Harris Hip Score

**Table 3:** P-LLD

	N Group (n = 55)	O Group (n = 48)	p
*Preoperative*			
P-LLD (%)	65.5% (36)	75.0% (36)	0.389
P-LLD cm (mean ± SD) [min–max]	0.71 ± 0.69 (0–2.0)	0.99 ± 1.07 (0–4.5)	0.112
*Postoperative*			
P-LLD (%)	25.5% (14)	58.3% (28)	0.001
P-LLD cm (mean ± SD) [min–max]	0.37 ± 0.87 (0–5.0)	0.91 ± 0.97 (0–3.0)	0.004
ΔP-LLD (mean ± SD) [min–max]	− 0.33 ± 1.14 (− 2.0 to 3.0)	− 0.08 ± 1.40 (− 2.5 to 1.0)	0.315

*P-LLD*, Perceived-leg length discrepancy

**Table 4:** Preoperative radiographic evaluations (spine)

	N group (n = 55)	O group (n = 48)	p
*Preoperative*			
Pelvic obliquity angle° (mean ± SD) [min–max]	2.5 ± 1.5 (0–6.3)	3.8 ± 2.3 (0–12)	0.001
Lumbar scoliosis angle° (mean ± SD) [min–max]	− 0.6 ± 6.4 (− 11 to 24)	0.1 ± 13.8 (− 41 to 54)	0.800
C7CVA mm (mean ± SD) [min–max]	− 0.9 ± 15.7 (− 38 to 41)	2.7 ± 17.8 (− 27 to 64)	0.280
LBR° (mean ± SD) [min–max]	9.3 ± 4.5 (0.6–17.6)	5.8 ± 4.7 (0–15.9)	< 0.001
PT° (mean ± SD) [min–max]	16.7 ± 8.9 (− 2.6 to 35.1)	13.4 ± 11.0 (− 13.3 to 38.3)	0.105
SS° (mean ± SD) [min–max]	34.5 ± 8.2 (15.9–51.3)	36.7 ± 8.2 (22.1–66.5)	0.179
PI° (mean ± SD) [min–max]	51.1 ± 9.0 (25.4–69.1)	50.1 ± 11.7 (25.0–84.5)	0.628
LL° (mean ± SD) [min–max]	35.0 ± 11.0 (1.5–57.7)	38.7 ± 14.2 (10.8–101)	0.136
PI-LL° (mean ± SD) [min–max]	16.1 ± 11.9 (− 9.8 to 51.6)	11.4 ± 15.3 (− 29.3 to 56.4)	0.081
TK° (mean ± SD) [min–max]	21.8 ± 10.9 (1.3–46.9)	22.7 ± 10.0 (2.8–43.1)	0.689
SVA mm (mean ± SD) [min–max]	28.9 ± 36.0 (− 39.6 to 132.7)	37.7 ± 41.3 (− 30 to 156)	0.254
*Postoperative*			
Pelvic obliquity angle° (mean ± SD) [min–max]	0.8 ± 0.6 (0–1.8)	4.0 ± 2.0 (2.0–11.1)	< 0.001

*C7CVA*, Cervical 7 coronal vertical axis; *LBR*, Lumber bending range; *PT*, Pelvic tilt; *SS*, Sacral slope. *PI*, Pelvic incidence; *LL*, Lumbar lordosis; *TK*, Thoracic kyphosis; *SVA*, Sagittal vertical axis

**Table 5:** Radiographic evaluations (Limb)

	N group (n = 55)	O group (n = 48)	p
*Preoperative*			
R-LLD mm (mean ± SD) [min–max]	9.0 ± 9.5 (− 13 to 37)	9.9 ± 12.1 (− 30 to 40)	0.661
Functional leg length discrepancy mm (mean ± SD) [min–max]	0.1 ± 8.1 (− 14 to 19)	1.7 ± 5.5 (− 37 to 22)	0.396
Femoral offset (operative side) mm (mean ± SD) [min–max]	38.0 ± 9.4 (4.1–58.3)	39.6 ± 8.7 (19.0–54.1)	0.402
Femoral offset (opposite side) mm (mean ± SD) [min–max]	41.4 ± 11.9 (11.0–53.0)	41.7 ± 7.7 (19.4–54.0)	0.902
Acetabular offset (operative side) mm (mean ± SD) [min–max]	106.0 ± 9.2 (81.7–134.4)	104.6 ± 9.8 (83.4–133.1)	0.476
Acetabular offset (opposite side) mm (mean ± SD) [min–max]	101.7 ± 11.6 (86.0–134.0)	101.1 ± 8.1 (87.0–123.0)	0.741
Global offset (operative side) mm (mean ± SD) [min–max]	144.0 ± 10.5 (120.5–174.0)	144.2 ± 10.8 (123.5–169.3)	0.933
Global offset (opposite side) mm (mean ± SD) [min–max]	143.1 ± 9.0 (120.0–164.0)	142.7 ± 9.5 (124.0–161.4)	0.821
*Postoperative*			
R-LLD mm (mean ± SD) [min–max]	0.1 ± 7.0 (− 24 to 15)	− 3.4 ± 10.3 (− 45 to 15)	0.048
Functional leg length discrepancy mm (mean ± SD) [min–max]	− 0.7 ± 5.8 (− 26 to 12)	2.4 ± 12.5 (− 25 to 50)	0.114
Femoral offset (operative side) mm (mean ± SD) [min–max]	43.3 ± 8.5 (17.9–61.7)	45.7 ± 9.8 (25.7–63)	0.195
Femoral offset (opposite side) mm (mean ± SD) [min–max]	41.5 ± 12.1 (11.0–54.0)	42.4 ± 6.6 (28.0–54.0)	0.644
Acetabular offset (operative side) mm (mean ± SD) [min–max]	101.9 ± 8.9 (82.3–123.6)	99.3 ± 7.2 (81.7–115.2)	0.106
Acetabular offset (opposite side) mm (mean ± SD) [min–max]	101.4 ± 11.4 (86.0–135.0)	100.6 ± 7.4 (87.0–124.0)	0.666
Global offset (operative side) mm (mean ± SD) [min–max]	145.2 ± 9.4 (118.1–164.7)	145.0 ± 12.3 (118.0–163.1)	0.899
Global offset (opposite side) mm (mean ± SD) [min–max]	142.9 ± 9.2 (118.0–164.0)	143.0 ± 9.5 (124.0–159.0)	0.967

*R-LLD*, Radiographic-leg length discrepancy

The risk factors for postoperative POA that were evaluated in the logistic regression analysis included age, sex, BMI, postoperative HHS activity, preoperative POA, and postoperative P-LLD. Age [odds ratio (OR) 0.957, 95% confidence interval (CI) 0.923–0.993, p = 0.020], preoperative POA (OR 1.490, 95% CI 1.100–2.020, p = 0.001), and LBR (OR 0.848, 95% CI 0.756–0.951, p = 0.005) were found to be significant factors influencing postoperative POA (Table 6).

**Table 6:** Logistic regression model for predicting postoperative pelvic obliquity ≥ 2°

	Odds ratio	95% CI	p
Age at THA (y)	0.957	0.923–0.993	0.020
Sex (male)	0.956	0.299–3.060	0.939
BMI	0.981	0.872–1.100	0.747
Postoperative HHS activity	0.888	0.711–1.110	0.292
Preoperative Pelvic obliquity (°)	1.490	1.100–2.020	0.010
LBR (°)	0.848	0.756–0.951	0.005
Postoperative R-LLD (mm)	0.501	0.244–1.030	0.059
Postoperative P-LLD (cm)	1.520	0.841–2.760	0.165

*PO*, Pelvic obliquity; *THA*, Total hip arthroplasty; *BMI*, Body Mass Index; *HHS*, Harris hip Score. *LBR*, Lumber bending range; *R-LLD*, Radiographic leg length discrepancy; *P-LLD*, Perceived-leg length discrepancy

## Discussion

In this study, we investigated the clinical outcomes and risk factors of patients presenting a POA ≥ 2° after THA. These patients had significantly lower HHS activity and total HHS and a higher prevalence of postoperative P-LLD. In addition, age, large preoperative POA, and LBR were identified as independent factors for a postoperative POA ≥ 2°.

It is unclear whether pelvic obliquity after THA affects the clinical outcomes. Takemoto et al. reported that the POA is an independent factor in the discrepancy between R-LLD and P-LLD in patients with preoperative osteoarthritis of the hip joint and that this discrepancy may affect hip function [5]. In the present study, patients with residual pelvic obliquity tended to have poor postoperative HHS and a large postoperative P-LLD. There is concern that residual pelvic obliquity following THA may have an impact not only on hip function but also on future adjacent joint disorders. As a result, surgeons must carefully plan surgeries to minimize the postoperative POA.

The POA after THA is expected to improve over time due to the correction of hip contracture and leg length difference [9]. Contrastingly, there are few documented cases of residual pelvic obliquity after THA, and there is no unified opinion regarding the factors involved [21]. Moharrami et al. reported that postoperative pelvic obliquity was correlated with preoperative pelvic obliquity and pre- and postoperative medial offset deviation [22]. The current study did not find medial offset to be a factor for residual postoperative pelvic obliquity, which may be because we performed accurate offset reconstruction based on preoperative planning using a 3D template, resulting in fewer cases of poor offset reconstruction [23]. In contrast, a large preoperative POA was identified as an independent factor in the present study. Therefore, careful preoperative planning is required when THA is performed in patients with a large POA, as there is a high risk that residual pelvic obliquity will remain postoperatively. However, contrary to our expectations, residual postoperative pelvic obliquity was more severe in younger patients. Severe developmental dysplasia of the hip often requires THA at a relatively young age [24]. Therefore, the longer disease duration since the onset of hip arthritis and the more severe degree of hip deformity in young patients with developmental dysplasia of the hip may have influenced the residual postoperative pelvic obliquity.

Poor spinal alignment is generally considered a factor affecting pelvic obliquity. Yu et al. reported that in cases of mild pelvic obliquity, the sacroiliac joint compensates, whereas when pelvic obliquity is severe, the lumbar spine and the sacroiliac joint compensate [3]. Furthermore, Koga et al. reported that patients with a large POA and poor lumbar spine mobility were more likely to experience P-LLD after THA [13]. However, it remains unclear how spinal parameters are linked to pelvic obliquity following THA. The results of the current study showed that sagittal alignment of the spine did not affect postoperative PO; however, poor LBR was an independent risk factor in patients with pelvic obliquity after THA. Even if THA improves hip contracture and leg length differences, in patients with poor LBR, pelvic obliquity may remain due to lumbar spine alignment.

It may be a primary concern of the surgeon to plan surgeries in attempt to avoid postoperative pelvic obliquity. After evaluating P-LLD, we believe that surgery should be performed to avoid as much leg length difference as possible in patients at high risk for residual pelvic obliquity. Nonetheless, reconstruction of hip function with THA alone is expected to have limitations in patients with severe lumbar spine range of motion. Consequently, when there is hip OA with large preoperative PO, it is critical to evaluate the lumbar spine and plan early surgical intervention.

The primary strength of this study is that it is the first to demonstrate the relationship between lumbar spine mobility and postoperative PO. Previous studies have shown that greater postoperative PO can lead to an increased perception of postoperative P-LLD in patients with similar radiographic leg length discrepancies, but they found no association between lumbar spine alignment and postoperative P-LLD. However, these studies did not evaluate lumbar spine mobility, and they suggested that lumbar spine mobility could be related to postoperative PO [25.26]. This novel finding elucidates the connection between P-LLD, lumbar spine mobility, and postoperative PO, bridging a gap in previous research.

This retrospective study had some limitations. First, the sample size was small (103 cases); a prospective evaluation of more cases is planned for the future. Second, the follow-up period was short (one year). Longer-term follow-up should be evaluated, since pelvic obliquity and the clinical outcomes of patients with residual pelvic obliquity may change over time.

## Conclusion

Patients with residual postoperative pelvic obliquity have poor hip function. Younger age, large preoperative POA, and poor lumbar spine mobility were identified as risk factors for residual postoperative pelvic obliquity.

## Data Availability

All data generated or analyzed during this study are included in this manuscript.
